# Racial Differences in Nicotine Reduction: Pooled Results from Two Double-Blind Randomized Controlled Trials

**DOI:** 10.1007/s40615-024-02155-1

**Published:** 2024-09-04

**Authors:** Wenxue Lin, Nicolle M. Krebs, Junjia Zhu, Kimberly Horn, Jonathan Foulds, A. Eden Evins, Joshua E. Muscat

**Affiliations:** 1 Department of Epidemiology and Biostatistics, College of Public Health, Temple University, Philadelphia, PA 19122 USA; 2 Department of Public Health Sciences, College of Medicine, Pennsylvania State University, Hershey, PA 17033 USA; 3 Department of Population Health Sciences, Virginia Tech Carilion Research Institute, Roanoke, VA 24016 USA; 4 Center for Addiction Medicine, Department of Psychiatry, Massachusetts General Hospital, Boston, MA 02114 USA; 5 Harvard Medical School, Boston, MA 02115 USA

**Keywords:** Racial differences, Reduced nicotine content, Usual nicotine content, Randomized controlled trial

## Abstract

**Introduction:**

Tobacco regulatory policies are generally intended to apply to all segments of the population and to be equitable. Results from clinical trials on switching from commercial cigarettes to reduced nicotine cigarettes have included black populations but race-specific findings are not widely reported.

**Methods:**

Data were pooled from two parallel randomized controlled trials of gradually reduced nicotine in cigarettes from 11.6 mg per cigarette down to 0.2 mg nicotine (very low nicotine content; VLNC) vs. usual nicotine content (UNC) cigarettes (11.6 mg) over an 18-week period in smokers with low socioeconomic status (SES) and mental health conditions, respectively. We used linear regression to determine the potential effects of cigarettes and biomarker reductions (blood cotinine and exhaled carbon monoxide) when using VLNC study cigarettes. An intention-to-treat (ITT) analysis included all randomized participants regardless of adherence to the protocol. A secondary compliance analysis compared control subjects (11.6 mg cigarettes) only to those switched to low nicotine cigarettes who were biochemically determined to be compliant to exclusively using VLNC cigarettes.

**Results:**

Both Black and White VLNC smokers had significantly lower plasma cotinine and exhaled carbon monoxide compared to those randomized to UNC cigarettes. The treatment × race interaction term was not significant for the outcome measures in both the ITT and secondary compliance analyses, except for cotinine in the ITT analysis (Whites: − 190 ng/mL vs. Blacks: − 118 ng/mL; *p* = 0.05).

**Conclusions:**

A reduced nicotine regulation for cigarettes would result in substantial reduction in exposure to nicotine and toxicants in Black and White smokers.

## Introduction

Tobacco use continues to be the leading cause of preventable mortality in the United States (U.S.) [1]. The U.S. Food and Drug Administration (FDA) released an advanced notice of proposed rulemaking in March 2018 aimed at reducing the permissible nicotine levels in cigarettes to minimally addictive or nonaddictive levels [2]. Commercially available cigarettes typically contain between 10 and 12 mg of nicotine. Levels of nicotine in cigarettes that are thought to be nonaddictive are about 1.0 mg or less. The White House supports the proposed rule to prevent the onset of nicotine addiction in youths and facilitate cessation. It also supports the rule for reducing disparities in nicotine dependence and advancing health equity [3]. Clinical studies conducted have provided consistent evidence that supports a nicotine product standard [4–10]. Randomized trials in smokers have demonstrated that switching directly to research cigarettes with very low nicotine content (VLNC) at 0.2 mg/cigarette or progressively reducing the nicotine content to 0.2 mg/cigarette gradually over several month reduced frequency of cigarette smoking, biomarkers of nicotine and tobacco smoke exposure, and cigarette dependence [6, 7]. Few adverse effects have been reported.

The 2009 Family Smoking Prevention and Tobacco Control Act refers to a Population Health Standard which defines the criteria for the regulation of tobacco products including the likelihood of long-term addictive use. A key component is that the benefits should be considered for the whole population. Tobacco products have historically been used more frequently in certain populations including some minority and ethnic groups, and in persons with low socioeconomic status and mental health conditions, and in co-users of other addicting agents [11]. Reporting research on the relative effects of traditional and alternative tobacco products in different risk groups for tobacco regulatory rulemaking has been advocated [11]. The effects of progressively reduced nicotine content (RNC) cigarettes in black smokers are not well characterized. Hatsukami et al. [12] compared immediate vs. progressive nicotine reduction down to VLNC levels. Reductions in biomarkers were found in both Black and White participants for both protocols. The current study pools data from two randomized trials in high-risk populations to examine the effects of nicotine reduction in black smokers and compare these effects to white smokers.

## Methods

We pooled data from two double-blind, two-arm, parallel-group randomized controlled trials of gradual nicotine reduction using research cigarettes formulated with nicotine at and below the levels in commercially available cigarettes. One trial was conducted at the Penn State University (PSU) College of Medicine (Hershey, PA, USA) and Massachusetts General Hospital (MGH) [13]. The other trial was conducted at the PSU College of Medicine (Hershey, PA, USA) and George Washington University (GWU) [7]. Both trials were conducted between 2015 and 2018. The PSU-MGH trial included smokers with mental health (MH) conditions that included mood and/or anxiety disorders, and the PSU-GWU clinical trial included cigarette smokers with low socioeconomic status (SES). Both MH and SES trials consisted of four phases (baseline 1, baseline 2, randomized, and treatment choice) with multiple clinic visits [for a total of 11 and 12 visits for PSU-GWU (SES sample) and PSU-MGH (MH sample), respectively] at the study centers over 33 weeks. Baseline phase1 included smoking participants’ usual brand cigarettes for 1 week. Baseline phase 2 included 2 weeks of smoking SPECTRUM usual nicotine content (UNC) study cigarettes (nicotine content approximately 11.6 mg/cigarette). In the 18-week randomized phase, participants were randomized to either the control arm in which they continued on the UNC study cigarettes, or the intervention arm to receive gradually reduced nicotine content (RNC) study cigarettes. Participants in the intervention arm received SPECTRUM cigarettes with nicotine contents of 7.4, 3.3, 1.4, 0.7, and 0.2 (VLNC) mg nicotine/cigarette (3 weeks per dose from 7.4 to 0.7 mg nicotine/cigarette, 6 weeks for 0.2 mg nicotine/cigarette) [7, 13, 14]. Cigarettes with 0.2 (VLNC) mg nicotine/cigarette are considered minimally or nonaddictive as the levels of nicotine are too low to provide reinforcing effects that sustain dependence [15].

Participants attended in-person visits during the randomization phase. Research coordinators collected blood samples and exhaled carbon monoxide (CO) readings to evaluate biomarkers of nicotine, toxicant exposure, and study compliance for both trials. Additional information about these two trials, such as study protocols, timeline, study flow diagrams, methods, randomization procedures, and primary outcomes were published elsewhere [7, 13, 14, 16]. All subjects provided written informed consent and the study was approved by the Massachusetts General Hospital, George Washington University and Penn State College of Medicine Institutional Review Boards. We developed methodologies for community-based recruitment of black smokers. This included economically disadvantaged individuals from urban areas [17]. Both studies are registered at clinicaltrials.gov (MH: NCT01928758; SES: NCT01928719).

### Measures

Demographic variables including race were obtained from eligible participants. Race response options were African American/black, Caucasian/White, American Indian/Alaskan native, Native Hawaiian/Pacific Islander, and other race. The current analysis was limited to Blacks and Whites as there were too few participants for meaningful statistical analyses from these latter groups.

Information on smoking behaviors and nicotine biomarkers was collected throughout the trial including cigarettes per day (CPD). Blood and exhaled breath CO readings were collected at each clinic visit (visit 3 to visit 9 for SES, visit 4 to visit 10 for MH). Participants were required to keep a daily cigarette log to record the number of study and non-study cigarettes smoked. We used an immunoassay kit and the piCO + Smokerlyzer to collect and assess plasma cotinine (ng/mL) and exhaled CO (ppm).

### Statistical Analyses

#### Pooled Analysis from SES and MH Randomized Controlled Trials

We determined the effect of race on outcome measures obtained at the last visit of the randomized phase (visit 9 and visit 10 for SES and MH, respectively). We measured and compared the treatment effects (defined as differences between VLNC and UNC groups) on plasma cotinine (ng/mL), CPD, and exhaled CO (ppm) outcome across the two racial groups after 6 weeks of smoking VLNC cigarettes at nicotine content of 0.2 mg nicotine/cigarette for both trials. Therefore, we included nicotine content treatment (VLNC or UNC) condition by race (Black and White) interaction term in the linear regression models to test whether race modifies the effects of VLNC. All linear regression models adjusted for baseline measure of the outcomes and covariates (study, age, gender, education, and employment status).

#### Primary Intention-to-Treat (ITT) Analysis vs. Secondary Compliance Analysis

The intention-to-treat (ITT) analysis, which evaluates participants based on their randomized treatment assignment regardless of their adherence to the assigned treatment, represents the gold standard framework for RCTs [18, 19]. It maintains the principle of randomization and offers a robust method to accurately evaluate the true impact of the treatment [18]. Our ITT analysis included a total of 403 Black and White smokers (*N*_Black_ = 104, *N*_White_ = 299).

A secondary compliance analysis was conducted that included the UNC group and only smokers from RNC group who were biochemically determined to be compliant to exclusively using VLNC cigarettes at the last study visit [20]. The compliance analysis included a total of 263 Black and White smokers (*N*_Black_ = 59, *N*_White_ = 204). Analyses were conducted using statistical software SAS Version 9.4 (SAS Institute Inc., Cary, NC, USA). All tests were two-sided and the significant level used was 0.05.

## Results

### Baseline Characteristics by Race

Table 1 presents demographic and baseline smoking characteristics in the Black and White smokers for SES and MH trials, respectively. At baseline, for the SES trial, Black smokers were older (50.9 vs. 41.2; *p* < 0.01) than White smokers. Black smokers reported smoking fewer CPD (21.5 vs. 25.4; *p* < 0.02) and had lower plasma cotinine (229.9 vs. 273.8; *p* < 0.05) and lower exhaled CO (22.6 vs. 36.3; *p* < 0.01) than White smokers. Further, most Black smokers (96.2%) chose menthol flavored cigarettes compared to 54.3% of White smokers (*p* < 0 . 01). There were significant differences in household income by race such that more than 70% of Black smokers had household income between $0 and $19,999 compared to 22.2% of White smokers (*p* < 0.01). For the MH trial, at baseline, Black smokers were older (47.9 vs. 42.7; *p* < 0.05), reported smoking fewer CPD (17.4 vs. 22.0; *p* < 0.01) and had lower exhaled CO (23.0 vs. 31.7; *p* < 0.02) than White smokers. Black smokers were more likely to use menthol cigarettes (76.9% vs. 34.9%; *p* < 0.01) and have a household income between $0-$19,999 (65.2% vs. 27.1%; *p* < 0.01) than White smokers.

**Table 1:** SES (visit 3) and MH (visit 4) baseline demographics by cigarette flavor

	SES			MH		
	Black smokers (*N* = 78)	White smokers (*N* = 153)	*p*-value	Black smokers (*N* = 26)	White smokers (*N* = 146)	*p*-value
Characteristics						
Age (years), mean (SD)	50.9 (7.6)	41.2 (11.7)	** < 0.0001**	47.9 (10.9)	42.7 (12.5)	**0.04**
Gender (%)			**0.013**			0.12
Female	33 (42.3)	91 (59.5)		12 (46.2)	91 (62.3)	
Male	45 (57.7)	62 (40.5)		14 (53.9)	55 (37.7)	
Cigarette flavor (%)			** < 0.0001**			** < 0.0001**
Menthol	75 (96.2)	83 (54.3)		20 (76.9)	51 (34.9)	
Non-menthol	3 (3.9)	70 (45.8)		6 (23.1)	95 (65.1)	
Hispanic (%)	0 (0.00)	3 (1.96)	0.12	1 (3.9)	2 (1.4)	0.37
Education (%)			0.13			0.56
Less than a HS degree or HS degree	43 (55.1)	100 (65.4)		11 (42.3)	53 (36.3)	
More than a HS degree	35 (44.9)	53 (34.6)		15 (57.7)	93 (63.7)	
Household Income (%)			** < 0.0001**			**0.0016**
$0–19,999	34 (70.8)	28 (22.2)		15 (65.2)	32 (27.1)	
$20,000–59,999	13 (27.1)	56 (44.4)		5 (21.7)	42 (35.6)	
$60,000 +	1 (2.1)	42 (33.3)		3 (13.0)	44 (37.3)	
Treatment (%)			0.21			0.36
Reduced nicotine	43 (55.1)	71 (46.4)		15 (57.7)	70 (48.0)	
Usual nicotine	35 (44.8)	82 (53.6)		11 (42.3)	76 (52.1)	
Cigarettes/day, mean (SD)	21.5 (10.2)	25.4 (13.5)	**0.016**	17.4 (6.5)	22.0 (11.3)	**0.0049**
Plasma cotinine (ng/mL), mean (SD)	229.9 (141.6)	273.8 (151.2)	**0.043**	277.4 (166.0)	255.4 (135.7)	0.47
Exhaled carbon monoxide, mean (SD)	22.6 (11.6)	36.3 (16.9)	** < 0.0001**	23.0 (13.0)	31.7 (16.8)	**0.013**

### Pooled Analysis of SES and MH Trials

Table 2 presents the pooled results from these two double-blind randomized controlled trials. Cotinine levels were significantly lower in the RNC group compared to the UNC group in both racial groups during the last visit of the randomized phase. Mean cotinine levels were reduced by (− 117.9 ng/mL for Blacks and − 190.0 ng/mL for Whites) in the ITT analysis, and (− 238.4 ng/mL for Blacks and − 274.8 ng/mL for Whites) in the secondary compliance analysis. Reductions in CPD (− 4.3 for Blacks and − 4.3 for Whites in the ITT) showed similar patterns in Black and White smokers. No significant interactions by race were observed for any of the outcomes in either the primary ITT or the secondary compliance analysis (*p*-value for interaction ≥ 0.05).

**Table 2:** Pooled results from two double-blind randomized controlled trials (primary ITT vs. secondary compliance analysis)

Outcome^1^	SES + MH				
	Black smokers		White smokers		
	*N* = 104 for ITT analysis *N* = 59 for compliance analysis		*N* = 299 for ITT analysis *N* = 204 for compliance analysis		Interaction
	Treatment effect (95% CI)	*p*-value	Treatment effect (95% CI)	*p*-value	*p*-value
Cotinine(ng/mL)					
Primary ITT analysis*	− 117.9 (− 179.2, − 56.5)	**0.0002**	− 190.0 (− 225.8, − 154.2)	** < 0.0001**	0.05
Compliance analysis^#^	− 238.4 (− 301.8, − 175.0)	** < 0.0001**	− 274.8 (− 308.2, − 241.3)	** < 0.0001**	0.32
Total CPD					
Primary ITT analysis	− 4.3 (− 8.0, − 0.6)	**0.02**	− 4.3 (− 6.6, − 2.0)	**0.0003**	0.98
Compliance analysis	− 4.1 (− 9.1, 1.2)	0.13	− 5.0 (− 7.9, − 2.1)	**0.0007**	0.76
Exhaled CO (ppm)					
Primary ITT analysis	− 4.3 (− 9.9, 1.21)	**0.13**	− 6.0 (− 9.5, − 2.5)	**0.0008**	0.61
Compliance analysis	− 2.3 (− 10.3, 5.7)	0.58	− 7.3 (− 11.7, − 2.9)	**0.0013**	0.27

*Treatment effect on outcomes at last visit of randomization phase by race: VLNC-UNC

^1^Adjusted for baseline measure of the outcomes, study, age, gender, education, and employment status

*Primary ITT analysis: participants are analyzed according to their randomized treatment assignment regardless of adherence to the assigned treatment

^#^Compliance analysis: only include participants who are complaint with VLNC cigarettes

## Discussion

The proposed tobacco product standard to set maximum nicotine level in cigarettes should be beneficial for the population as a whole and advancing health equity in reducing disparities in the risk of nicotine addiction and tobacco-related diseases [21]. Blacks who smoke typically have a higher cotinine per cigarette ratio and elevated mortality rates of many smoking-related diseases compared to White smokers [12, 22–24].

The current study determined the effect of switching from fully nicotinized cigarettes to VLNC in both Blacks and Whites who currently smoke cigarettes using pooled results from two randomized trials with similar study designs. Overall, both Black and White participants randomized to RNC presented significantly lower plasma cotinine, exhaled carbon monoxide, and smoking behavior compared to smokers of UNC. We found that race did not modify the effect of reduced nicotine content cigarettes, as indicated in the primary ITT analyses (*p*-value for interaction ≥ 0.05).

Our RCT trials were conducted to determine the effect of a reduced nicotine rule for cigarettes in a scenario where usual brand cigarettes with normal nicotine content are not legally available in the market. However, participants randomized to receive VLNC cigarettes were living in an environment where normal nicotine cigarettes were freely available. It is therefore not surprising that some participants did not adhere to the exclusive use of VLNC, and supplemented with nicotine from other sources (i.e., their preferred commercial brand of cigarettes). However, if the FDA implements a reduced nicotine standard policy, smokers would have legal access solely to VLNC cigarettes [18]. Some smokers might acquire illicit UNC cigarettes from the black market, and so the primary ITT analysis could accurately reflect the effects of a VLNC cigarette standard [18]. In the compliance analysis where we exclusively compared the effect of VLNC between smokers who adhered to the treatment assignment, the interaction term for race was not significant. For the SES trial, the quit rate was 9/122 (7%) in the RNC group and 5/123 (4%) in the UNC group. In the MH trial, 17/94 (18.1%) of the RNC group and 4/94 (4.3%) of the UNC group met the criteria for abstinence. In the SES trial, among the 62 participants assessed for biochemical compliance at the end of the randomized phase, 43.5% (27/62) were fully compliant, and 56.5% (35/62) were noncompliant. In the MH trial, 59.4% (41/69) of the RNC group met self-report and biochemical criteria for strict adherence. It should also be noted that in these trials participants were asked not to use alternative nicotine products (e.g., electronic cigarettes) and very few reported such use. In a national implementation of a “reduced nicotine cigarette policy,” it would be expected that many smokers would use non-smoked nicotine products.

The current study had some limitations. First, all study cigarettes were provided free of charge and participants received monetary compensation and stipends to reimburse them for their travel to the clinics. Thus, our results may not reflect the real-world situation of smokers’ product use behavior if RNC cigarettes become the only cigarettes legally available [12]. A second limitation of our study is that we only measured cotinine levels and did not measure total nicotine equivalent (TNE) levels. More Black than White smokers are slow nicotine metabolizers. Thus, total nicotine equivalents are the best measure for racial comparisons when comparing nicotine exposure. Our analysis did not include other races such as American Indian/Alaskan native and Native Hawaiian/Pacific Islander due to their limited sample size. Given the limited sample size and variability among racial minority participants, there may be insufficient power to detect a significant interaction effect on exhaled CO toxic smoke exposure. This limitation complicates our ability to conclude that VLNC manipulation effectively reduces a cardiovascular toxin in cigarette in cigarette smoke exposure in this important subgroup of Black participants.

## Conclusion

Both Black and White VLNC smokers presented significantly lower plasma cotinine and smoking behavior compared to usual nicotine content (UNC) cigarette smokers. While the reduction on exhaled CO was significant in White VLNC smokers but not Black VLNC smokers, the interaction was not significant. Race did not moderate the effect of reduced nicotine content cigarettes on most outcomes in both the primary ITT and secondary compliance analyses, except for whites achieving a greater reduction in cotinine in the ITT analysis. A reduced nicotine standard for cigarettes would likely achieve substantial reductions in nicotine and smoke toxicants in Blacks and Whites. The current studies were not specifically designed for cessation purposes. Tobacco policies need to consider alternatives to low nicotine cigarettes to reduce harm from smoking if users of low nicotine cigarettes do not quit even when the cigarettes are non-addicting. Because these cigarettes still have reinforcing value, they should not be marketed to youths who are experimenting with cigarettes. Public health messages to youths need to emphasize that low nicotine cigarettes are still toxic and that they may lead to regular smoking despite their low nicotine content. Further work in this area would help inform both policies and tobacco control initiatives.

## Data Availability

Data cannot be shared publicly. The study participants did not consent to having their data shared publicly.
